# Supplementary material for: MetaQTL: a package of new computational methods for the meta-analysis of QTL mapping experiments
Source: BMC Bioinformatics. 2007 Feb 8;8:49. doi: 10.1186/1471-2105-8-49 (PMC1808479; doi:10.1186/1471-2105-8-49)
Supplement: Additional File 7 — MetaQTL Package : jar file and tutorial. This Zip archive contains both the MetaQTL JAR file and the files of the tutorial. [file 1471-2105-8-49-S7.zip › org.inra.metaqtl/doc/constant-values.html]

Constant Field Values


|  |  |  |
| --- | --- | --- |
| **Overview** \| Package \| Class \| Use \| **Tree** \| **Deprecated** \| **Index** \| **Help** | |  |
| PREV   NEXT | **FRAMES**    **NO FRAMES**     **All Classes** |  |


---


# Constant Field Values


---

**Contents**

- org.metaqtl.\*

org.metaqtl.\*

| org.metaqtl.EMCriteria | | |
| --- | --- | --- |
| `public static final int` | `AIC` | `0` |
| `public static final java.lang.String` | `AIC_NAME` | `"AIC"` |
| `public static final int` | `AIC3` | `1` |
| `public static final java.lang.String` | `AIC3_NAME` | `"AIC3"` |
| `public static final int` | `AICc` | `5` |
| `public static final java.lang.String` | `AICc_NAME` | `"AICc"` |
| `public static final int` | `AWE` | `4` |
| `public static final java.lang.String` | `AWE_NAME` | `"AWE"` |
| `public static final int` | `BIC` | `3` |
| `public static final java.lang.String` | `BIC_NAME` | `"BIC"` |
| `public static final int` | `ICOMP` | `2` |
| `public static final java.lang.String` | `ICOMP_NAME` | `"ICOMP"` |

| org.metaqtl.IBioGenomeProperties | | |
| --- | --- | --- |
| `public static final java.lang.String` | `CROSS_SIZE` | `"cross.size"` |
| `public static final java.lang.String` | `CROSS_TYPE` | `"cross.type"` |
| `public static final java.lang.String` | `MAPPING_CROSS` | `"mapping.cross"` |
| `public static final java.lang.String` | `MAPPING_EXPANSION` | `"mapping.expansion"` |
| `public static final java.lang.String` | `MAPPING_FUNCTION` | `"mapping.function"` |
| `public static final java.lang.String` | `MAPPING_FUNCTION_HALDANE` | `"haldane"` |
| `public static final java.lang.String` | `MAPPING_FUNCTION_KOSAMBI` | `"kosambi"` |
| `public static final java.lang.String` | `MAPPING_UNIT` | `"mapping.unit"` |
| `public static final java.lang.String` | `MAPPING_UNIT_CM` | `"cM"` |
| `public static final java.lang.String` | `MAPPING_UNIT_M` | `"M"` |

| org.metaqtl.IBioLGroupProperties | | |
| --- | --- | --- |
| `public static final java.lang.String` | `CHISQUARE` | `"meta.chisquare"` |
| `public static final java.lang.String` | `DOF` | `"meta.dof"` |
| `public static final java.lang.String` | `PVALUE` | `"meta.pvalue"` |

| org.metaqtl.IBioLocusProperties | | |
| --- | --- | --- |
| `public static final java.lang.String` | `DUBIOUS_ORDER` | `"dubious.order"` |
| `public static final java.lang.String` | `OCCURENCE` | `"meta.occurence"` |
| `public static final java.lang.String` | `QTL_CI_FROM` | `"qtl.ci.from"` |
| `public static final java.lang.String` | `QTL_CI_LOD_DECREASE` | `"qtl.ci.lod.decrease"` |
| `public static final java.lang.String` | `QTL_CI_TO` | `"qtl.ci.to"` |
| `public static final java.lang.String` | `QTL_CROSS_NAME` | `"qtl.cross.name"` |
| `public static final java.lang.String` | `QTL_CROSS_SIZE` | `"qtl.cross.size"` |
| `public static final java.lang.String` | `QTL_CROSS_TYPE` | `"qtl.cross.type"` |
| `public static final java.lang.String` | `QTL_PROJ_INTSCALE` | `"qtl.proj.intScale"` |
| `public static final java.lang.String` | `QTL_PROJ_MAPSCALE` | `"qtl.proj.mapScale"` |
| `public static final java.lang.String` | `QTL_PROJ_ORIGIN` | `"qtl.proj.mapOrigin"` |
| `public static final java.lang.String` | `QTL_PROJ_SHAREFLANKING` | `"qtl.proj.shareFlanking"` |
| `public static final java.lang.String` | `QTL_PROJ_SWAPFLANKING` | `"qtl.proj.swapFlanking"` |
| `public static final java.lang.String` | `QTL_STATS_ADDITIVE` | `"qtl.effect.additive"` |
| `public static final java.lang.String` | `QTL_STATS_DOMINANCE` | `"qtl.effect.dominance"` |
| `public static final java.lang.String` | `QTL_STATS_RSQUARE` | `"qtl.rsquare"` |
| `public static final java.lang.String` | `QTL_TRAIT_GROUP` | `"qtl.trait.group"` |
| `public static final java.lang.String` | `QTL_TRAIT_NAME` | `"qtl.trait.name"` |
| `public static final java.lang.String` | `QTL_TRAIT_UNIT` | `"qtl.trait.unit"` |

| org.metaqtl.IMetaQtlConstants | | |
| --- | --- | --- |
| `public static final int` | `CI_MISS_IGNORE` | `2` |
| `public static final int` | `CI_MISS_IMPUTATION` | `1` |
| `public static final int` | `CI_MODE_AVAILABLE` | `1` |
| `public static final int` | `CI_MODE_LOD` | `2` |
| `public static final int` | `CI_MODE_MAXIMAL` | `4` |
| `public static final int` | `CI_MODE_RSQUARE` | `3` |
| `public static final int` | `MAPPING_FUNCTION_HALDANE` | `1` |
| `public static final int` | `MAPPING_FUNCTION_KOSAMBI` | `2` |
| `public static final int` | `MAPPING_FUNCTION_UNKNOWN` | `0` |
| `public static final int` | `MAPPING_UNIT_CM` | `2` |
| `public static final int` | `MAPPING_UNIT_M` | `1` |
| `public static final int` | `MAPPING_UNIT_UNKNOWN` | `0` |
| `public static final double` | `TINY_MAPPING_DISTANCE` | `1.0E-5` |
| `public static final double` | `TINY_MAPPING_VARIANCE` | `1.0E-32` |
| `public static final java.lang.String` | `VERSION` | `"1.0"` |

| org.metaqtl.QtlPartition | | |
| --- | --- | --- |
| `public static final int` | `HORIZONTAL_MODE` | `0` |
| `public static final int` | `VERTICAL_MODE` | `1` |

| org.metaqtl.algo.EMAlgorithm | | |
| --- | --- | --- |
| `public static final int` | `EM_CONTINUE` | `1` |
| `public static final int` | `EM_FAILURE` | `2` |
| `public static final int` | `EM_OK` | `0` |
| `public static final double` | `TINY_Z_PROBA` | `4.9E-324` |

| org.metaqtl.algo.HClustAlgorithm | | |
| --- | --- | --- |
| `public static final int` | `CENTROID_METHOD` | `1` |
| `public static final int` | `WARD_METHOD` | `2` |

| org.metaqtl.bio.IBioCrossTypes | | |
| --- | --- | --- |
| `public static final int` | `BC` | `1` |
| `public static final int` | `DH` | `6` |
| `public static final int` | `IRISe` | `7` |
| `public static final int` | `IRISi` | `8` |
| `public static final int` | `RF` | `5` |
| `public static final int` | `RILSe` | `2` |
| `public static final int` | `RILSi` | `3` |
| `public static final int` | `SF` | `4` |
| `public static final int` | `UNKNOWN` | `0` |

| org.metaqtl.bio.IBioEntityTypes | | |
| --- | --- | --- |
| `public static final int` | `ALLELE` | `8` |
| `public static final int` | `COLLECTION` | `9` |
| `public static final int` | `GENOME` | `3` |
| `public static final int` | `INDIVIDUAL` | `1` |
| `public static final int` | `LGROUP` | `4` |
| `public static final int` | `LOCUS` | `5` |
| `public static final int` | `MARKERLOCUS` | `6` |
| `public static final int` | `ONTOLOGY` | `11` |
| `public static final int` | `ONTOLOGY_TERM` | `10` |
| `public static final int` | `POPULATION` | `2` |
| `public static final int` | `QTLLOCUS` | `7` |

| org.metaqtl.bio.IBioLocus | | |
| --- | --- | --- |
| `public static final int` | `MARKER` | `0` |
| `public static final int` | `QTL` | `1` |

| org.metaqtl.bio.IOntologyTermProperties | | |
| --- | --- | --- |
| `public static final java.lang.String` | `DEFINITION` | `"term.definition"` |
| `public static final java.lang.String` | `SYNONYMS` | `"term.synonyms"` |

| org.metaqtl.bio.entity.IGeneticMapProperties | | |
| --- | --- | --- |
| `public static final java.lang.String` | `cartoFunctionHaldane` | `"haldane"` |
| `public static final java.lang.String` | `cartoFunctionKey` | `"carto.function"` |
| `public static final java.lang.String` | `cartoFunctionKosambi` | `"kosambi"` |
| `public static final java.lang.String` | `distanceUnitCM` | `"cM"` |
| `public static final java.lang.String` | `distanceUnitKey` | `"distance.unit"` |

| org.metaqtl.bio.util.ILGroupPosition | | |
| --- | --- | --- |
| `public static final int` | `ESTIMATED` | `2` |
| `public static final int` | `STATIC` | `1` |

| org.metaqtl.factory.MetaQtlModelFactory | | |
| --- | --- | --- |
| `public static final java.lang.String` | `SEPARATOR` | `"\t"` |

| org.metaqtl.graph.ChromLayer | | |
| --- | --- | --- |
| `public static final int` | `ALIGN_FIRST_COMMON` | `0` |
| `public static final int` | `ALIGN_FIRST_MARKER` | `1` |
| `public static final int` | `LEFT_LABEL` | `1` |
| `public static final int` | `NEG_COMMON` | `1` |
| `public static final int` | `POS_COMMON` | `0` |
| `public static final int` | `RIGHT_LABEL` | `0` |
| `public static final int` | `SINGLE_COMMON` | `2` |

| org.metaqtl.graph.ChromShape | | |
| --- | --- | --- |
| `public static final int` | `LEFT_LABEL` | `1` |
| `public static final int` | `RIGHT_LABEL` | `0` |

| org.metaqtl.util.OffspringManager.HaldaneWaddingtonProportion | | |
| --- | --- | --- |
| `public static final double` | `C_coeff` | `1.0` |
| `public static final double` | `D_coeff` | `1.0` |
| `public static final double` | `E_coeff` | `2.0` |
| `public static final double` | `F_coeff` | `0.5` |
| `public static final double` | `G_coeff` | `0.5` |

---


|  |  |  |
| --- | --- | --- |
| **Overview** \| Package \| Class \| Use \| **Tree** \| **Deprecated** \| **Index** \| **Help** | |  |
| PREV   NEXT | **FRAMES**    **NO FRAMES**     **All Classes** |  |


---
